# Feline Neural Progenitor Cells I: Long-Term Expansion under Defined Culture Conditions

**DOI:** 10.1155/2012/108340

**Published:** 2012-04-23

**Authors:** Jing Yang, Jinmei Wang, Ping Gu, X. Joann You, Henry Klassen

**Affiliations:** ^1^Department of Ophthalmology, Ophthalmology Research Laboratories, The Gavin Herbert Eye Institute, University of California, Irvine, CA 92697, USA; ^2^Department of Ophthalmology, Shanghai Ninth People's Hospital, School of Medicine, Shanghai Jiaotong University, Shanghai 200011, China

## Abstract

Neural progenitor cells (NPCs) of feline origin (cNPCs) have demonstrated utility in transplantation experiments, yet are difficult to grow in culture beyond the 1 month time frame. Here we use an enriched, serum-free base medium (Ultraculture) and report the successful long-term propagation of these cells. Primary cultures were derived from fetal brain tissue and passaged in DMEM/F12-based or Ultraculture-based proliferation media, both in the presence of EGF + bFGF. Cells in standard DMEM/F12-based medium ceased to proliferate by 1-month, whereas the cells in the Ultraculture-based medium continued to grow for at least 5 months (end of study) with no evidence of senescence. The Ultraculture-based cultures expressed lower levels of progenitor and lineage-associated markers under proliferation conditions but retained multipotency as evidenced by the ability to differentiate into neurons and glia following growth factor removal in the presence of FBS. Importantly, later passage cNPCs did not develop chromosomal aberrations.

## 1. Introduction

The mammalian central nervous system (CNS) has a restricted capacity for self-repair and regeneration and, as a consequence, the extent of clinical recovery from CNS injury or disease is generally limited. Because of this unmet clinical need, much work has been devoted to exploring potential ways of enhancing clinical outcomes in the setting of debilitating neurological conditions. One particularly interesting approach is the transplantation of allogeneic neural progenitor cells (NPCs). These multipotent cells are derived from the developing nervous system and, under defined serum-free conditions, are capable of at least limited expansion in culture, followed by differentiation into mature neurons and glia, either following cessation of mitogenic stimulation in vitro or transplantation to the diseased CNS [1, 2].

There are a number of characteristics of NPCs that recommend them for application to neural repair strategies. From a practical standpoint, the proliferative capability of the cells mentioned above allows for the generation of cell banks [3], thereby decreasing the need for continued derivation from donor tissue. From a biological standpoint, the ability of NPCs to exhibit directed migration to areas of disease and integrate into the local cytoarchitecture represents a major breakthrough compared to previous work, for example, with fetal tissue transplantation [4, 5]. From a clinical standpoint, the immune tolerance-afforded allogeneic NPCs in animal studies [6–8] would appear to obviate the need for mandatory immune suppression in many cases, hopefully including allografts in humans. If this is the case, it would substantially decrease the therapeutic risk to patients. In addition, it would appear that progenitor cells of this type convey a substantially decreased risk of tumor formation, particularly when compared to analogous cells derived from pluripotent cell types [9].

In addition to their potential role in cell replacement, NPCs also represent an attractive method for gene delivery, particularly with respect to neuroprotective cytokines. These molecules are gene products that are rapidly degraded in vivo by endogenous proteases and notably include trophic factors such as glial cell line-derived neurotrophic factor (GDNF). It has been demonstrated that NPCs can be genetically modified to express these types of factors ex vivo, expanded in number, and subsequently transplanted for study [10, 11]. Given that these cells are typically well tolerated immunologically, genetically modified NPCs could provide a method of sustained drug delivery to local sites within the brain, retina, and spinal cord. This option is of interest in species, where NPCs can be successfully isolated, and where models of CNS disease are available.

Progenitor or precursor cells have now been isolated and grown from viable brain tissue in a broad range of mammalian species, including mouse, rat, cat, pig, sheep, dog, monkey, and human [12]. The cat represents a model of interest for neural repair strategies because of the potential for detailed electrophysiological and behavioral studies. In previous work, we and another group have reported the isolation of feline neural progenitor-like cells, combined with successful transplantation to the dystrophic retina [13] and the normal brain [14] of allorecipients. Nevertheless, it has proven difficult to grow these cells for extended periods in culture using conventional protocols. This lack of in vitro expansion hampers further research by restricting the number of studies that can be performed from a given isolation. Here, we identify a modified culture method that allows for sustained, abundant growth of feline neural progenitor cells sufficient for banking. We provide additional characterization of these cells, including examination of karyotype and analysis of gene expression at multiple time points in culture.

## 2. Materials and Methods

### 2.1. Isolation and Culture of NPCs from the Cat (cNPCs)

The isolation of cNPCs followed a protocol similar to that described previously [13], but in this case the donor was a 47-day timed-pregnant domestic cat (E47). Fetuses were removed under terminal anesthesia at an academic veterinary facility and shipped on ice to the site of cell isolation and culture. Upon arrival, brains were removed by dissection and the forebrain separated from the cerebellum and brainstem. Forebrain tissue was relocated to a petridish containing cold DMEM (Invitrogen, Carlsbad, CA, USA). The tissue was minced using 2 scalpels and then enzymatically digested using 0.05% TrypLE Express (Invitrogen). The resulting cell suspension was washed repeatedly and dissociated by repeated, gentle aspiration using a flame-polished glass pipette. The resulting cells were then divided and seeded into 1 of 2 different complete culture media, namely, standard medium (SM) or Ultraculture-based medium (UM). SM consisted of Advanced DMEM/F12 (Invitrogen), 1% by volume N2 neural supplement (Invitrogen), 1% GlutaMax (Invitrogen), 50 U/mL penicillin-streptomycin (Invitrogen), 20 ng/mL epidermal growth factor (recombinant human EGF, Invitrogen) and 20 ng/mL basic fibroblast growth factor (recombinant human bFGF; Invitrogen). To promote adherence, 5% FBS (Sigma) was also included at the time of initial plating. The following day all medium in cultures was completely replaced with serum-free SM. The remaining half of the primary cells were seeded in an alternate proliferation medium, henceforth designated UltraCulture-based medium (UM), containing Ultraculture (Lonza), 1% GlutaMax, 50 U/mL penicillin-streptomycin, 20 ng/mL epidermal growth factor, and 20 ng/mL basic fibroblast growth factor. The plating density was 0.5 × 10^6^ cells/mL for both conditions. Subsequently, cells were fed by medium exchange every 2 to 3 days and passaged at confluence using TrypLE Express and gentle trituration through a flame-polished glass pipette. At each passage, cell number was counted using a hemocytometer.

### 2.2. Cytogenetic Analysis (Chromosome Counting and Karyotyping)

Confluent cNPCs generated in UM were harvested at culture passages 8 and 14 and prepared for analysis, as follows. Cells were plated onto a T-25 flask, and the media were changed 24 hours before harvesting the culture to stimulate cell division and maximize the mitotic index. The cells were then mitotically arrested with colcemid (KaryoMax Colcemid solution, Invitrogen) at a final working concentration of 0.12 *μ*g/mL at 37°C for 20 minutes. Isolated cNPCs were harvested for hypotonic treatment in 0.075 M KCl solution at room temperature for 25 minutes. The cells were pelleted by centrifugation at 1000 rpm for 8 minutes and fixed in ice-cold fixative (3 : 1 methanol: glacial acetic acid) for 10 min. After a second wash in fixative, the cells were resuspended in 2 mL fixative. Slides were prepared by dropping the cell suspension onto dry microscope slides prewashed with fixative. G-banded karyotyping was performed by Cell Line Genetics LLC (Madison, WI, USA).

### 2.3. RNA Isolation and cDNA Synthesis

Total RNA was extracted from passage 8 cNPCs using the RNeasy Mini Kit (Qiagen, Valencia, CA, USA) according to the manufacturer's protocol. DNase I was used to digest and eliminate any contaminating genomic DNA. Two micrograms of total RNA in a 20 *μ*L reaction were reverse-transcribed using an Omniscript cDNA Synthesis Kit (Qiagen, Valencia, CA, USA).

### 2.4. Quantitative PCR (qPCR)

qPCR was performed on an Applied Biosystems 7500 Fast Real-Time PCR Detection System (Applied Biosystems, Foster, CA, USA) following protocols previously described [15]. Briefly, 20 *μ*L total reaction was made up of 10 *μ*L 2x Power SYBR Green PCR Master Mix (Applied Biosystems, Foster, CA, USA), 100 ng cDNA, and gene specific primers (Table 1) at a concentration of 300 *μ*M. Samples were initially denatured at 95°C for 10 min, followed by 40 cycles of PCR amplification (15 seconds at 95°C and 1 minute at 60°C). To normalize template input, *β*-actin (endogenous control) transcript level was measured for each sample. Melting curves were determined to confirm amplification of the expected fragment. Fold change and heat map were generated using JMP 4.1 and DataAssist 2.0 software.

### 2.5. Induction of cNPC Differentiation

The cNPCs were cultured in UM or UM-FBS that contained 10%FBS but no added growth factors with the same cell density, 0.3 × 10^6^ cells/mL. Culture media was exchanged every 2 days. The morphology of cells was monitored every day, and the cells were photographed on days 1, 3, 5, and 7.

### 2.6. Immunocytochemistry

After 4 months in culture, cNPCs were plated on four-well chamber slides in either UM or UM-FBS medium and fed every two days. On day 5, cells were fixed with freshly prepared 4% paraformaldehyde (Invitrogen) in 0.1 M phosphate-buffered saline (PBS) for 20 minutes at room temperature. Fixed cells were washed with PBS, then they were incubated for 1 hour at room temperature in antibody blocking buffer containing the following: PBS containing 10% (v/v) normal donkey serum (Sigma), 0.3% Triton X-100, and 0.1% NaN3 (Sigma-Aldrich, Saint Louis, MO, USA). Slides were then incubated in primary antibodies (Table 2) at 4°C overnight. After washing, slides were incubated for 1 hour at room temperature in fluorescent-conjugated secondary antibody, 1 : 400 Alexa Fluor^546^ goat anti-mouse, followed by washings. Cell nuclei were counterstained with 1.5 *μ*g/mL 4′,6-diamidino-2-phenylindole (DAPI; Invitrogen, Molecular Probes, Eugene, OR, USA) in Vectashield Hard Set Mounting Medium (Vector Laboratories, Burlingame, CA, USA) for 15 min at room temperature. Negative controls for immunolabeling were performed in parallel using the same protocol but with omission of the primary antibody. Fluorescent labeling was judged as positive only with reference to the negative controls. Immunoreactive cells were visualized and images recorded using a Nikon fluorescent microscope (Eclipse E600; Nikon, Melville, NY, USA).

## 3. Results

### 3.1. Comparison of cNPCs Grown in Different Proliferation Media

**Figure 1:**
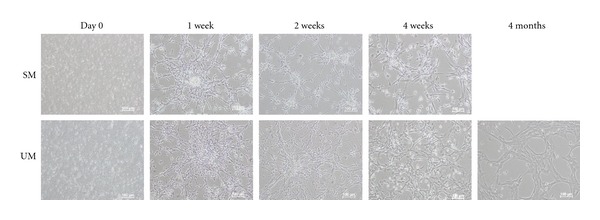
Morphology of cNPCs in different proliferation media at serial time points. Upper panel shows cNPCs cultured in SM and the lower panel cells of the same age in UM. Cultures are shown on day 0, after 1, 2, and 4 weeks as well as 4 months (UM only). In both media the cells exhibited morphologic features consistent with primitive neural cells throughout the period examined. In both cases, the adherent cells extended processes and expanded greatly during the initial week. The cells in SM ceased expanding during the first month and were not evaluated beyond the 3-month time point, whereas those in UM continued to expand throughout the course of the study. Scale bar: 100 *μ*m.

Proliferative cultures were obtained from forebrain-derived feline NPCs seeded and maintained in both types of media (SM and UM); however, only those seeded in UM continued to expand throughout the duration of the study (5 months). The cells in both types of media exhibited morphologic features consistent with primitive neuroectodermal cells throughout their growth period (Figure 1). In both cultures, the majority of cells were adherent to the surface of the flask and continued to proliferate, forming a pattern of random networks and nodal clusters as is typical of mammalian neural progenitors when not grown as suspended neurospheres.

The morphology of cNPCs cultured in the two different growth media was also examined. In both conditions, the initially dissociated cells divided and formed small clusters over the first week in culture. During this period, cellular processes had started to form by day 3 and were greatly elaborated by the end of the first week. Cells cultured in SM showed little evidence of proliferation beyond week 3, but continued to survive up to 3 months. In contrast, cells cultured in UM established stably expanding populations. The cNPCs continued to expand vigorously, while maintaining progenitor morphology, although a tendency of the cells to enlarge and flatten was observed (Figure 1).

### 3.2. Long-Term Expansion of cNPC Cells Is Possible in UM

**Figure 2:**
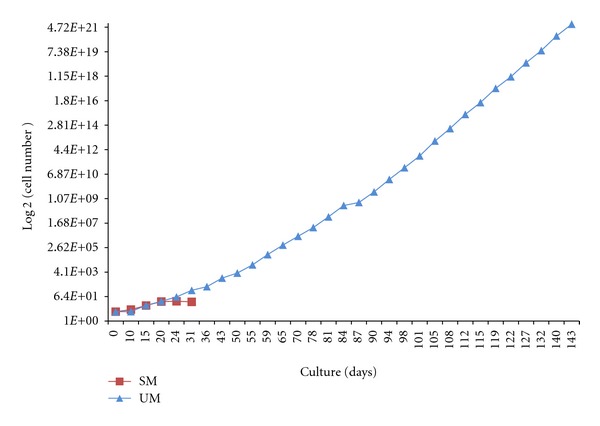
Expansion capacity of cNPC cells in long-term culture. Cells were cultured in 1 of 2 proliferation media of differing composition and counted at each passage using a hemocytometer. The number of cells in SM medium (red) increased initially, then began to drop shortly after day 20, with no measurable proliferation beyond 1 month. In contrast, cultures grown in UM (blue) exhibited sustained growth throughout the course of the study (143 days), with no evidence of senescence. The rate of expansion did not diminish with passage number. Numbers on *x*-axis represent days in culture at each passage, the *y*-axis shows cell number as total estimated yield.

The growth characteristics of cNPCs are plotted in Figure 2. Initial growth of cells was observed in either medium, but sustained expansion was only achieved using UM. The number of cells in SM medium increased initially and peaked shortly after day 20. After that, the total cell count began to drop, and no further passaging or counting was performed although the cells continued to survive up to at least 3 months. In contrast, the cNPCs in UM continued to increase steadily, without indications of senescence, throughout the 5-month duration of this study.

### 3.3. Cytogenetics of cNPCs during Extended Culture

**Figure 3:**
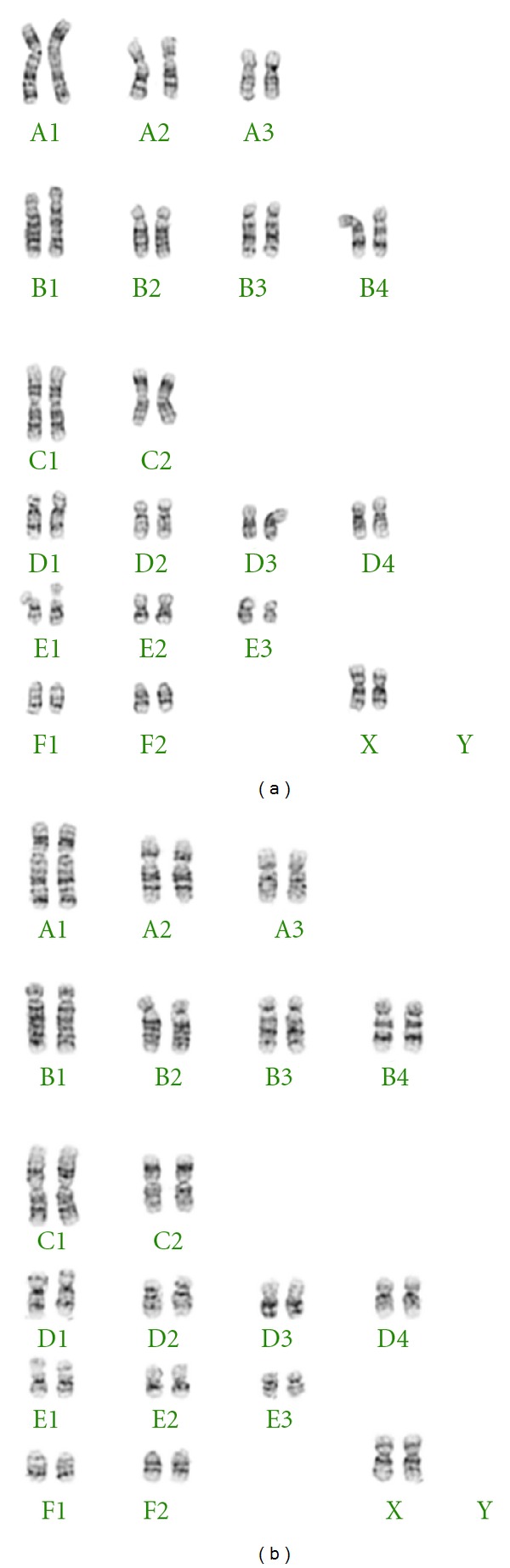
G-banded karyotyping of cNPCs at early and later passage. Cytogenetic G-banded karyotyping results based on analysis of 20 metaphase cNPCs. (a) passage 8 (day 50); (b) passage 14 (day 87). The cells from both time points exhibited a normal 38XX feline karyotype.

Because increased rates of cellular proliferation can be the result of chromosomal abnormalities arising during extended periods of cell culture, the karyotypic stability of cNPCs cultures in UM was evaluated by chromosome counting and G-banded karyotyping. Cytogenetic analysis was performed on twenty G-banded metaphase cells from passage 8 (day 50) and from passage 14 (day 87) time points that roughly corresponded to possible upward inflections in the growth curve. The results showed that the cells from both time points possessed normal feline 38XX karyotypes (Figure 3), indicating that the increased proliferation rate seen was not the result of a culture-induced chromosomal abnormality.

### 3.4. Quantitative Evaluation of the Effect of Different Culture Media on cNPC Gene Expression

**Figure 4:**
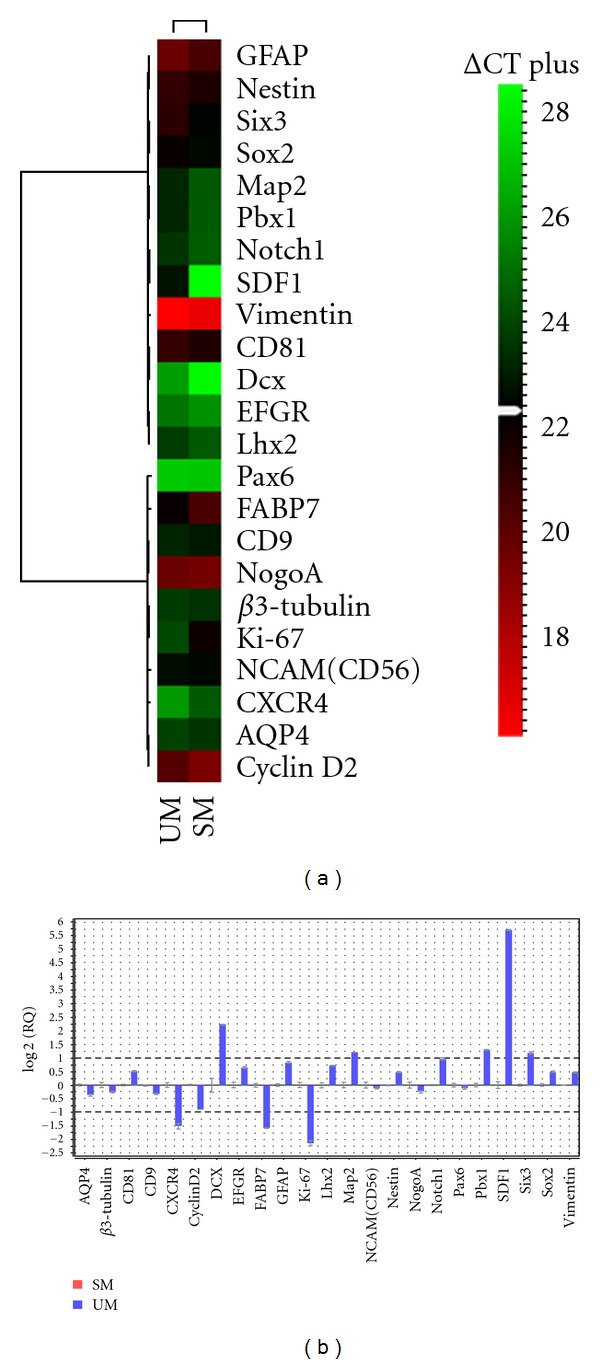
Comparison by qPCR of gene expression profile for cNPCs in different culture media. The cNPCs were cultured in either SM or UM for 1 month prior to testing. (a) heat map display showing relative expression levels of 23 genes expressed by NPCs or related progeny of neural lineage. The scale to the right shows that moderate expression level (as determined by CT value) is shown as black, while lower expression appears increasingly green, and higher expression increasingly red. Viewed in this way, the general similarity of the 2 conditions is evident in that the color of a particular gene tends to be conserved across conditions, even if the intensity often varies. (b) fold change, with SM used as calibrator. Note that expression is represented on a log2 scale, such that 1.00 corresponds to a 2 fold change. Viewed in this way, differences in expression are highlighted. The majority of genes showed less than 2 fold change (between 1.00 to −1.00, on log2 scale), again confirming the general similarity between conditions; however, a number of individual genes fell outside this range. The error bars-show standard deviation.

Having determined that UM effectively sustains the proliferation of NPCs of feline origin, whereas the conventional media formation did not, it was of interest to compare the phenotype of the cells grown using these methods. In order to look for differences related to the alternate proliferation conditions used, we compared gene expression profiles for cNPCs grown in SM versus UM at the 1 month time point using quantitative RT-PCR (Figures 4(a) and 4(b)).

Both sampled populations expressed the neural progenitor-associated genes nestin, sox-2, vimentin, and notch1 as well as the proliferation markers cyclinD2 and Ki-67 (Figure 4(a)). Expression of the markers CD81 and FABP7, which are also known to be expressed by NPCs [16–18], was detected as well. Low expression of the early neuronal marker *β*-III tubulin was seen, as reported previously [13]. The mature neuronal and astroglial markers Map2 and GFAP were detectable, the latter more prominently than the former. Overall, these results were consistent with the maintenance of markers associated with neural progenitor populations by cNPCs when grown in UM, including the modest but detectable tendency for ongoing, spontaneous differentiation along the neuronal lineage, as previously reported in analogous cells from various mammalian species.

Looked at more closely, there were similarities and differences in the level of expression for particular markers (Figure 4(b)). Less than 2 fold variance between conditions was observed for expression of the majority of markers including AQP4, *β*3-tubulin, CD9, CD81, CyclinD2, EGFR, GFAP, Lhx2, NCAM, nestin, nogoA, notch1, Pax6, Sox2, and vimentin. Growth in UM was associated with greater than 2 fold increased expression in the neuroblast marker DCX, the neuronal marker Map2, the transcription factor Pbx1, and the migration-associated marker SDF1. Markers that were greater than 2 fold lower in UM were CXCR4, FABP7 and Ki-67. Of note, the most upregulated marker (SDF1) is a receptor for the migration factor CXCR4, which was downregulated.

### 3.5. Sequential Analysis of cNPC Gene Profile with Time in Culture

**Figure 5:**
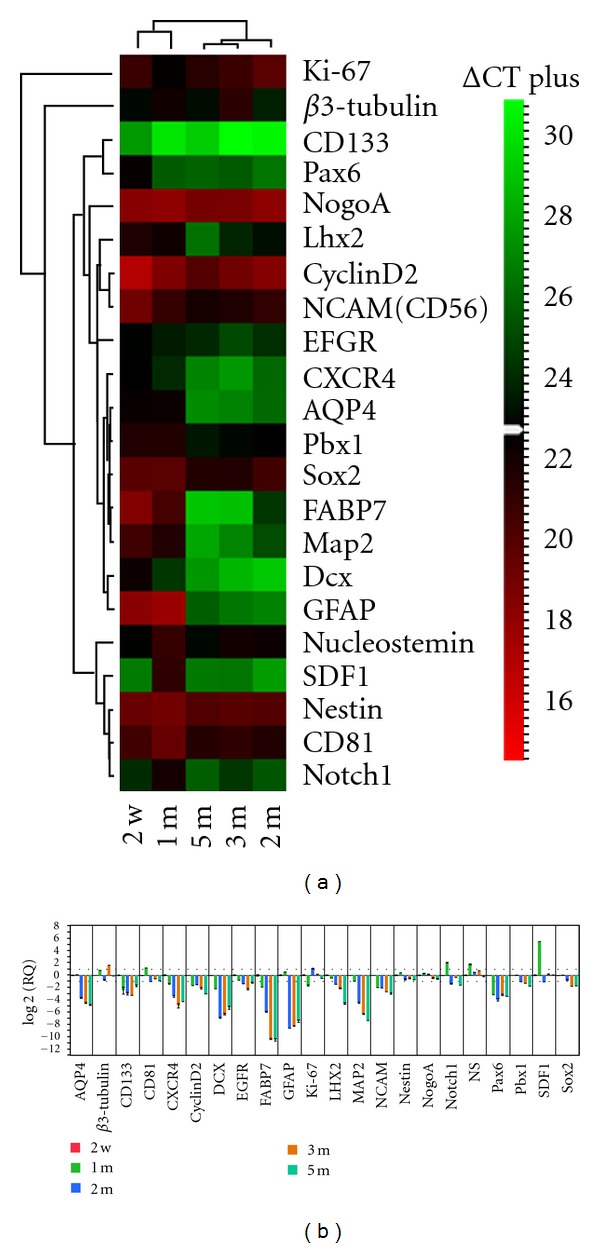
Comparison of gene expression from cNPCs at different time points in UM via qPCR. A 22-gene profile was assayed at each time point. The initial time point for comparison was 2 weeks (calibrator), followed by 1, 2, 3 and 5 months. (a) viewed as a heat map, there was an apparent overall drop in marker expression (shift from red towards green end of spectrum), and this was most evident between the 1- and 2-month time points, both visually and by cluster analysis (dendrogram at top). (b) data viewed as RQ (fold change) confirms the prevalent downward trend with time as well as highlighting the quantitative differences in fold change between markers. Error bars show standard deviation.

To examine the phenotypic stability of cRPCs during extended culture in UM, we employed qPCR, in this case to compare the expression profile obtained at 2 weeks to that present at 1, 2, 3, and 5 months (Figure 5). A preponderance of the markers examined showed a tendency to decrease with time in culture. This trend included progenitor-associated and neurodevelopmental markers as well as some markers associated with further lineage restriction. Both heat map and cluster analysis indicated an overall drop in marker expression between the 1- and 2-month time points (Figure 5(a)). Interestingly, this is the same time frame in which the cells in UM diverged in growth characteristics from those in SM, as seen above in Figure 2. Viewed as a histogram, the qPCR data showed a sequential downward trend, although expression levels appeared to be leveling off at the latter time points (Figure 5(b)). Again, this is concomitant to the robust proliferation seen beyond 1 month in the UM condition.

### 3.6. Differentiation

**Figure 6:**
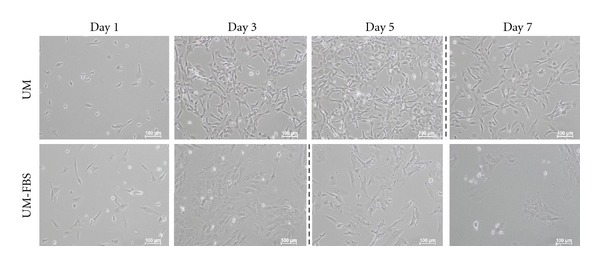
Morphology of cNPCs grown under proliferation versus differentiation conditions. Cultured cNPCs grown under proliferation conditions (UM), beginning at P26 (4 months), maintained the appearance of neural progenitors, while those grown under differentiation conditions (UM-FBS) appeared similar but exhibited a more flattened morphology. Dotted vertical lines represent passaging/reseeding, thereby accounting for the decreased cell density in images to the right of those points. Cells in the UM-FBS condition reached confluence more quickly.

Having determined that cNPCs can be grown beyond the 1-month time point using UM, it was important to confirm whether cNPCS grown in this medium retain the potential to differentiate into both neuronal and glial cell types. As a first step, cNPCs (4 months, P26) were dissociated into single cells and induced to differentiate by culture in UM without growth factors, but containing 10% serum (UM-FBS), for 7 days. The cells cultured in UM-FBS appeared similar but exhibited a more flattened morphology than undifferentiated controls (Figure 6). Interestingly, the cultures in UM-FBS reached confluence more rapidly than those in UM. The next step was to examine the expression of relevant markers.

### 3.7. Effect of UM-FBS on Marker Expression of cNPCs, Evaluated by Immunocytochemistry

**Figure 7:**
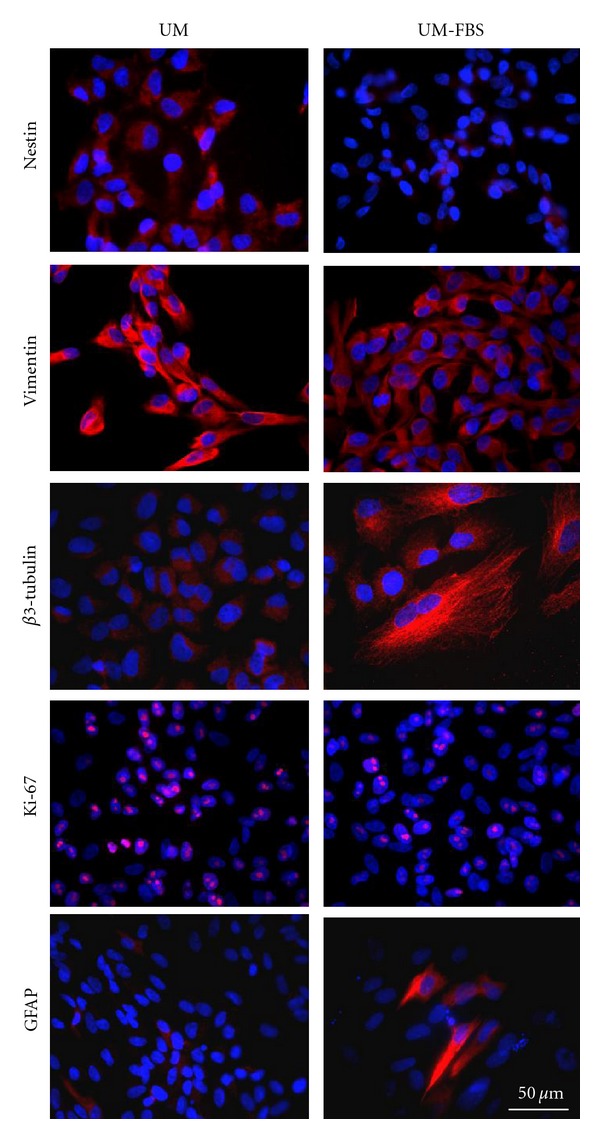
Effect of differentiation conditions on expression of markers by immunocytochemistry. To examine the lineage potential of cNPCs, P26 (4 month) cultures were grown in UM or UM-FBS for 5 days and immunolabeled with antibodies (red) against nestin, vimentin, *β*3-tubulin, Ki-67, and GFAP. Cell nuclei were labeled with DAPI (blue). Scale bars represent 50 *μ*m.

Immunocytochemical analysis (Figure 7) at the same time point (4 months, P26) confirmed that feline cells cultured in UM expressed a numbers of markers associated with neural precursor cells. These included strong expression of the intermediate filaments nestin and vimentin as well as the proliferation marker Ki-67. There was also trace labeling for the lineage markers *β*3-tubulin and GFAP, both of which are cytoskeletal proteins. *β*3-tubulin is a marker of neurons, and GFAP is strongly expressed by astrocytes. These data are suggestive of a small but detectable level of spontaneous differentiation in the cultures under proliferation conditions, as is expected with cells of this type.

At 5 days after induction of differentiation in UM-FBS, very few cells remained nestin positive, the signal for vimentin persisted at a diminished level, and expression of Ki-67 had decreased notably. In contrast, many more cells were positive for *β*3-tubulin and a subset of cells GFAP was strongly positive for GFAP, consistent with differentiation along neuronal as well as glial lineages (Table 3).

## 4. Discussion

Since their initial isolation, neural progenitor cells have been viewed as a powerful research tool for experimental investigation of novel approaches to cell replacement throughout the central nervous system (CNS). The recognized potential of NPC transplantation-based regenerative therapy for CNS diseases has generated considerable enthusiasm among many investigators and resulted in rapid growth of the field. The scientific understanding of NPCs has increased accordingly, although transplantation of these cells has yet to achieve accepted clinical use. One challenge has been growing sufficient quantities of the cells, and this is, therefore, a fundamental area deserving of additional attention. Refinement and the optimization of culture conditions is an obvious initial approach to further sustaining the proliferation of NPCs *in vitro*. It is also important to consider that the culture requirements of progenitor cells may differ between species. Research has shown that extended culture of neural progenitors is often associated with loss of multipotency, particularly a reduced potential to generate neurons, together with loss of self-renewal, as reflected in a marked propensity towards early senescence [19, 20]. A pertinent issue is the extent to which changes in culture conditions might enhance the expansion of functionally multipotent NPCs.

Feline NPCs can be difficult to propagate using conventional serum-free conditions. Here, we directly compared two variations on serum-free proliferation media, SM and UM, which differed in base medium but contained the same growth factors. Both formulations were used to examine their ability to sustain the proliferation and development of cNPCs derived from E47 brain tissue. In the more conventional SM medium, cNPCs stopped dividing and began to senesce by 1-month in culture. In UM, the cells continued to exhibit vigorous growth for up to 5 months, the latest time point examined, thereby allowing the banking of considerable numbers of mitotically active cNPCs. Although cNPCs grown in SM and UM appear similar in terms of certain key genes expressed, quantitative analysis of expression level did reveal differences between the conditions at the 1 month point. Interestingly, the expression level of the majority of genes, including progenitor and lineage markers, was downregulated in UM versus SM. Furthermore, this tendency toward downregulation in UM was even more pronounced beyond the 1-month time point, although the possible trend toward a new, lower set point in expression was noted. The reason for this is not clear, but might relate to a state of continuous, rapid cell division. What is clear is that UM is strongly permissive of feline NPCs survival and proliferation *in vitro*, whereas use of conventional SM medium rapidly leads to a failure to propagate.

One possible explanation for the facile growth exhibited by cNPCs in UM could be spontaneous immortalization. The cells in UM displayed repeated upward inflections in growth rate with time in culture that might reflect dysregulation of the cell cycle. It is known that immortalization of NPCs can occur with extended time in culture, and that such events are frequently associated with abnormalities in karyotype. To examine this, we evaluated whether karyotypic alterations were present in our cells, and the results showed that despite 14 passages in UM, the karyotype remained stable. Therefore, the improved growth seen in UM cannot be attributed to changes in karyotype.

Because altered gene expression might be associated with a loss of multipotency, it was important to confirm whether NPCs grown in UM maintain their ability to differentiate into cells of neural lineage. Comparing various progenitor markers and differentiation markers in UM versus UM-FBS conditions, we found that expression levels of progenitor markers decreased while neuronal and glial markers increased. These data indicate that cNPCs cultured in UM retain multipotency and the capacity to differentiate.

The source of the improvement in growth of cBPCs seen in UM thus appears to reside in the beneficial effects of the media constituents rather than aberrations of cellular proliferation. There is no question that Ultraculture is a much richer base medium, containing approximately 6 fold greater total protein than SM. While it is tempting to speculate that certain serum proteins or peptides may be critical to the growth of feline progenitors, further work is needed to define which particular components are responsible for the dramatic improvements seen in the current study.

In summary, we have shown that the use of a highly enriched, serum-free medium allows the long-term propagation of feline neural progenitor cells, something that standard serum-free conditions does poorly, if at all. The resulting cells retain multipotency and the ability to differentiate, as well as a normal karyotype. This does not rule out the possibility that the cells may have taken a significant, but less obvious, step towards spontaneous immortalization, and that the growth-promoting influence of UM might be causative. Fortunately, the resulting ability to generate and bank large numbers of cNPCs should greatly facilitate additional examination of these cells, including both safety concerns and the potential for therapeutic benefits following transplantation.

## Figures and Tables

**Table 1:** Cat-specific primers for real-time PCR.

Genes	Description	Forward (5′–3′)	Reverse (5′–3′)	Annealing temperature (°C)	Product size (base pairs)
Nestin	Intermediate filament, Progenitor	CTGGAGCAGGAGAAGGAGAG	GAAGCTGAGGGAAGCCTTG	60	180
Sox-2	Transcription factor, progenitor	ACCAGCTCGCAGACCTACAT	TGGAGTGGGAGGAAGAGGTA	60	154
Vimentin	Intermediate filament, progenitor	ATCCAGGAGCTACAGGCTCA	GGACCTGTCTCCGGTACTCA	60	247
Pax 6	Transcription factor, progenitor	AGGAGGGGGAGAGAATACCA	CTTTCTCGGGCAAACACATC	60	183
Notch1	Surface receptor, progenitor	CAGTGTCTGCAGGGCTACAC	CTCGCACAGAAACTCGTTGA	60	231
CD133	Progenitor	AGGAAGTGCTTTGCGGTCT	TGCCAGTTTCCGAGTCTTTT	60	120
Cyclin D2	Cell cycle protein	CAAGATCACCAACACGGATG	ATATCCCGCACGTCTGTAGG	60	162
Ki-67	Cell cycle protein, proliferation	TCGTCTGAAGCCGGAGTTAT	TCTTCTTTTCCCGATGGTTG	60	150
CD81	Tetraspaniin	CCACAGACCACCAACCTTCT	CAGGCACTGGGACTCCTG	60	156
EGFR	EGF receptor, surface marker	AACTGTGAGGTGGTCCTTGG	CGCAGTCCGGTTTTATTTGT	60	231
FABP7	fatty acid binding protein	TGGAGGCTTTCTGTGCTACC	TGCTTTGTGTCCTGATCACC	60	165
*β*3-tubulin	Microtubule protein, neural precursor	CATTCTCGTGGACCTTGAGC	GCAGTCGCAATTCTCACATT	60	199
Map2	Microtubule-associated, neuron	ACCTAAGCCATGTGACATCCA	CTCCAGGTACATGGTGAGCA	60	152
GFAP	Intermediate filament, glia	CGGTTTTTGAGGAAGATCCA	TTGGACCGATACCACTCCTC	60	188
AQP4	water channel protein	TACACTGGTGCCAGCATGA	CACCAGCGAGGACAGCTC	60	118
SDF 1	stromal-cell-derived factor-1	ACAGATGTCCTTGCCGATTC	CCACTTCAATTTCGGGTCAA	60	152
CXCR4	fusin	TCTGTGGCAGACCTCCTCTT	TTTCAGCCAACAGCTTCCTT	60	220
Dcx	Doublecortin, neuroblast marker	GGCTGACCTGACTCGATCTC	GCTTTCATATTGGCGGATGT	60	222
Lhx2	Homeobox transcription factor	GATCTGGCGGCCTACAAC	AGGACCCGTTTGGTGAGG	60	224
NCAM (CD56)	Adhesion molecule, surface marker	AGAACAAGGCTGGAGAGCAG	TTTCGGGTAGAAGTCCTCCA	60	172
NogoA	Reticulon 4, surface protein	TTTGCAGTGTTGATGTGGGTA	TAACAGGAACGCTGAAGAGTGA	60	100
nucleostemin	Nucleolar protein	CAGTGGTGTTCAGAGCCTCA	CCGAATGGCTTTGCTGTAA	60	165
Pbx 1	Transcription factor	CTCCGATTACAGAGCCAAGC	GCTGACCATACGCTCGATCT	60	166
*β*-actin	Housekeeping gene	GCCGTCTTCCCTTCCATC	CTTCTCCATGTCGTCCCAGT	60	168

**Table 2:** Primary antibodies for immunocytochemistry.

Antigen	Host species and reactivity in retina	Dilution	Source
Nestin	Mouse monoclonal; progenitors, reactive glia	1 : 200	BD, 611658
Vimentin	Mouse monoclonal; progenitors, reactive glia	1 : 200	Sigma, V6630
*β*III-tubulin	Mouse monoclonal; immature neurons	1 : 200	Chemicon, MAB1637
Ki-67	Mouse monoclonal; proliferating cells	1 : 200	BD, 556003
GFAP	Mouse monoclonal; Astrocyte, reactive glia	1 : 200	Chemicon, MAB3402

**Table 3:** Estimated percentage and intensity of labeling of cultured cNPCs for specific markers after 5-day exposure to differentiation conditions (UM-FBS). +: weak expression; ++: moderate expression; +++: strong expression.

	Nestin	Vimentin	*β*3-tubulin	Ki-67	GFAP
UM	100/++	100/+++	60/+	85/++	5/+
UM-FBS	2/+	100/++	85/++	60/+	35/+++

## Abbreviations

AQP4: Aquaporin 4
FABP7: Fatty acid binding protein 7
GFAP: Glial fibrillary acidic protein
NCAM: Neural cell adhesion molecule
SDF1: Stromal cell-derived factor 1
RT-PCR: Reverse transcriptase-polymerase chain reaction.
